# Lower limb prosthesis abandonment and recycling of used components in the public healthcare sector of the Eastern Cape Province of South Africa

**DOI:** 10.33137/cpoj.v9i1.47107

**Published:** 2026-04-29

**Authors:** X.L. Goxo, S. Visagie, L. Ned

**Affiliations:** 1 Division of Disability and Rehabilitation Studies, Faculty of Medicine and Health Sciences, Stellenbosch University, Cape Town, South Africa.; 2 Department of Medical Orthotics and Prosthetics, Faculty of Health Sciences, Durban University of Technology, KwaZulu Natal, South Africa.

**Keywords:** Lower Limb Prosthesis, Public Healthcare, Prosthetic Components, Prosthesis Abandonment, Amputation, Eastern Cape, Prosthetic Services, Prosthesis, South Africa

## Abstract

**BACKGROUND:**

Waiting times for lower limb prosthesis in the public healthcare sector of South Africa can exceed 12-months. A cause of long waiting times is shortage of prosthetic components. However, sometimes prostheses are abandoned. Recycling components from unused prostheses to mitigate the shortage of components has not been studied.

**OBJECTIVE:**

This study aimed to determine the reasons for lower limb prosthesis abandonment and to explore the possibility of recovering unused prostheses for component recycling in the public healthcare sector of the Eastern Cape Province, South Africa.

**METHODOLOGY:**

A cross-sectional survey was conducted among individuals who had stopped using or had an unused lower limb prosthesis. Participants were recruited using total population sampling from three public hospitals with Orthotics and Prosthetics centres in the Eastern Cape. During routine follow-up (July–August 2025), 92 individuals who had received a lower limb prosthesis between January 2021 and January 2025, or their next of kin where applicable, were contacted. Individuals still using their prosthesis and children (<18 years) were excluded. The remaining 45 non-users or next of kin were invited to participate. A self-developed structured questionnaire was administered via short (10–15 minute) interviews, and data were analyzed descriptively using SPSS (version 31).

**FINDINGS:**

A total of 43 participants aged 18–77 years (median 54; interquartile range 24), including 54% (n=23) male participants, consented to participate and completed the interviews. Twenty-two participants (51%) reported poorly fitting sockets as the reason for non-use. Forty-two (98%) participants were in favour of recycling components. Forty (93%) of the unused prostheses were modular. The majority were transtibial (81%), followed by transfemoral (12%) and knee disarticulation (7%). The main barrier to recycling identified was damaged components (19%). Thirty-nine (91%) prostheses were recovered from which 190 components can possibly be recycled.

**CONCLUSION:**

The findings extend existing knowledge on prosthesis abandonment and highlight an opportunity to implement component recycling practices to improve access, reduce waiting times, and lower costs in low-resource settings with high prosthetic service demand. The study may have important implications for prosthetic service delivery and policy in the Eastern Cape and South Africa.

## INTRODUCTION

It is estimated that 35 to 40 million people across the world require prosthetic devices,^1^ and Stevens^2^ suggested that roughly 30 million of these people live in low-income countries. In low-and-middle income countries (LMIC), such as South Africa, prosthetic services users are faced with complex service access barriers. These barriers include environmental factors like poor transportation access coupled with long distances, and rural living.^3–6^ From the side of service delivery, a shortage of manpower, materials, and components add to prosthetic service challenges.^7–9^ Collectively these barriers often lead to prosthetic waiting times of a year or longer, and sometimes persons with amputation requiring a prosthesis do not receive one at all.^10–11^ Not having a prosthesis increases dependency and impacts negatively on the mobility, socioeconomic participation and health related quality of life of persons with amputation.^12,13^

A study conducted in 2018 in the public healthcare sector of the Eastern Cape province of South Africa reported an accumulative prosthetic waiting list of more than 600 persons.^14^ In 2024, the Eastern Cape Department of Health financial report indicated that 1,276 people were waiting for a prosthesis.^15^ These figures show an increase of 113% over five years. To aggravate matters, lower limb amputation is on the increase internationally,^8,16^ and in South Africa driving an increasing demand for prosthetic services.^17^

One of the causes of long waiting times is the shortage of prosthetic components accompanied by troubled supply chain processes.^4–9^ South African clinicians observed that some lower limb prostheses were returned by the next-of-kin or the user either because the user has passed away or the prosthesis was abandoned. However, this observation has never been studied, and numbers of returned prostheses were not quantified. Roffman et al.^18^ reported a lower limb prosthesis non-use prevalence rate of 18% (n = 102), 12-months post discharge from rehabilitation, in Australia. A more recent narrative review by Piscitelli et al.,^19^ that included nine studies, reported a prosthesis abandonment rate ranging between 24% to 70%.

The reasons for non-use of lower limb prostheses were described by Roffman et al.^20^ as declining physical strength, residual limb surgical review, emotional factors, and/or death. Paquette et al.^21^ in a systematic review and meta-analysis, reported that reasons for lower limb prosthesis abandonment broadly include user-related factors (e.g., comorbidities and lack of motivation) and prosthesis-related factors (e.g., poorly fitting sockets, difficulty in donning, and the need for device replacement). Poorly fitting prostheses were also reported by Pienaar and Visagie^11^ in South Africa and Turner et al.^22^ in England, as a reason for prosthesis abandonment. Other reasons for non-use of lower limb prosthesis reported by Valgeirsdóttir^23^ were high maintenance, abandoning the prosthesis for a less sophisticated device, inability to use the prosthesis due to heavy weight, fear of falling and phantom pain.

Unused prostheses can potentially be recovered and re-introduced into the pool of available devices through recycling.^24^ Recovering unused prostheses for component recycling was identified internationally where Non-Governmental Organizations (NGOs) from high-income countries such as the United States collect and donate unused prostheses to users in LMIC.^25^ For prosthetic components to be re-used it should be in good condition,^26^ as guided by Standard 20 of the World Health Organization standards for prosthetic services, which state that “reuse of prosthetic and orthotic components should be regulated by a designated authority or group of experts with no conflict of interests and involve proper quality control and documentation”.^1^ Unused assistive products can be retrieved in various ways including users returning them to health facilities, follow-up home visits or outreach programs to re-assess the usage of assistive products and retrieval if unused. Additional options include toll-free telephone numbers, word-of-mouth, newsletters, tradeshows, advertising on company vehicles, websites and places of worships.^27–34^

There is existing evidence of lower limb prosthesis abandonment in the Eastern Cape. Similarly, evidence exists of recycling donated prosthetic components in LMICs. Diverse strategies have been proposed for recovering unused assistive products for re-use. However, there is a need for more context specific research in this area. This study, therefore evolved to answer the questions, what are the reasons for abandonment of lower limb prostheses and what is the possibility of recovering unused prostheses to recycle components in the public healthcare sector of the Eastern Cape Province of South Africa? This study aimed to determine the reasons for non-use of lower limb prostheses, obtain the opinions of prostheses non-users on the adoption of a prosthetic components recycling practice and their willingness to return the unused prostheses for recycling. Finally, it aimed to determine the number of lower limb prosthetic components that can possibly be recycled. The novelty of this study is to examine the feasibility of integrating prosthetic component recovery into existing prosthetic service delivery in the Eastern Cape Province of South Africa. It may provide a health systems-level perspective that is underrepresented in the current literature.

## METHODOLOGY

### Study Design and Participant Selection

A cross-sectional survey was conducted among individuals with lower limb amputation, or their next of kin, who received a prosthesis between 1 January 2021 and 31 January 2025 and were no longer using it or had an unused prosthesis. Participants were recruited using total population sampling from three hospitals with Orthotics and Prosthetics (O&P) centers in the Eastern Cape, which are the sole providers of public prosthetic services in the province. During routine follow-up (28 July–22 August 2025), clinical staff contacted 92 individuals who had been fitted with lower limb prostheses (transfemoral, knee disarticulation, or transtibial), or their next of kin where applicable.

Beeson et al.^35^ reported that individuals with amputation due to vascular disease have a high five-year mortality rate. In addition, contact information for individuals who received a prosthesis more than five years earlier may no longer be accurate or available. Individuals who had received their prosthesis less than six months prior to follow-up were excluded, as they may still have been in the adaptation phase of prosthesis use. These considerations were used to justify the selected time frame for the study.

During follow-up, one of the questions was whether participants were still using their prosthesis. Clinical staff informed non-users and next of kin in possession of unused prostheses about the study. In cases where users had died, next of kin were informed and invited to participate.

Subsequently, 47 individuals were excluded: 39 adults who were still using their prosthesis, seven children under the age of 18 years, and one next of kin. The remaining 45 individuals (49%), comprising 42 prosthesis non-users and three next of kin, were informed about the study and asked about their interest in participation. Those who expressed interest were then asked to provide consent for their contact details to be shared with the first author (Goxo X.L).

### Data Collection Procedures

The data collection tool was developed by the first author (Goxo X.L), an experienced prosthetist. It was reviewed by the second (Visagie S) and third authors (Ned L), both of whom have experience in prosthetic rehabilitation and survey research, to establish face and content validity. Further validation was supported through testing with two individuals with lower limb prostheses who had previously stopped using their prosthesis. The participants reported that the questions were clear and comprehensive and did not suggest any modifications to the survey.

The survey (**Appendix A**) consisted of 19 close and open-ended questions, structured to cover the participants’ demographic information, amputation and prosthetic related information, reasons for non-use, and their opinions on returning and recycling of prostheses components. The survey was completed during face-to-face interviews at the hospital or participants’ homes, depending on their choice. It took around 10 to 15 minutes to complete. Data was collected in IsiXhosa or English which are the two most common languages in the Eastern Cape. The choice of language was according to the participant’s preference. After completion of the survey, the participants willing to return the unused prosthesis gave it to the first author who took it to the providing hospital for storage. The first author entered all data onto an Excel spreadsheet. After a completeness check, which showed no missing data, 10% of the data was checked for correctness.

The data was analyzed through descriptive statistical analysis using SPSS (version 31). Median and interquartile range (IQR) were used for numerical data. Categorical data was summarized with frequencies. Open-ended responses were coded by assigning numerical values to similar responses within each survey question category. The categories were developed inductively, and the coded data were then analyzed quantitatively. The first and second authors familiarized themselves with the data after which they developed inductive coding categories together, following a consultative process. The first author coded the data. Codes were verified by the second author.

The relatively small sample size and the use of a survey tool that was not tested for construct validity may have reduced internal validity. The sample size was lower than the required 96 participants needed to achieve a 10% margin of error with a 95% confidence interval, assuming an unknown population size. Therefore, the findings should be interpreted and generalized with caution. The self-developed tool was not tested for reliability; however, data were collected by a single researcher using a standardized approach, thereby eliminating interrater variability. Finally, potential researcher bias was minimized, as the first author had no affiliation with the participating hospitals and had not provided prosthetic services to any of the participants.

### Study setting

The Eastern Cape Province covers an area of 168,966 km² and has a population of approximately 7.2 million people.^36^ It is among the least resourced provinces in South Africa, with many residents living in remote areas, which limits access to healthcare services.^9^ More than 90% of the population relies on public healthcare services.^15^

Prosthetic services are provided by 23 prosthetists.^9^ In 2023, the three hospitals that are the sole providers of public prosthetic services in the province received a total of 1,607 prosthesis applications, while providing 331 prostheses at an average cost of seven thousand rand (approximately USD 390) per prosthesis.^15^ The remaining 1,276 applicants were placed on a waiting list.

### Ethical Considerations

Ethical approval for this study was obtained from Stellenbosch University Health Research Ethics Committee (S24/07/184 (PhD) and permission to conduct the study in the Eastern Cape was granted at provincial and hospital level (EC_202412_024). The privacy and confidentiality of participants were maintained as required by the Protection of Personal Information Act (POPIA).^37^

## RESULTS

### Participant Characteristics

Of the 45 people identified with unused lower limb prosthesis, 43 agreed to participate of which, three (3) were next-of-kins of deceased prosthesis users. Participants’ ages ranged from 18 to 77 years (Median = 54, Interquartile Range = 24 years). Twenty-three participants (54%) identified as male. **Table 1** shows that the level of amputation was mostly transtibial (35; 81%). The most common cause of amputation was trauma (21; 49%). Forty participants (93%) were fitted with modular prostheses.

**Table 1:** Participant Demographics and Prosthesis Information.

Variable	Category	Participants (n)	Percentage (%)*
**Causes of amputation**	Trauma	21	49
	Diabetes mellitus	10	23
	Infections	6	14
	Congenital deformities	4	9
	Vascular disease	1	2
	Cancer	1	2

**Type of prosthesis**	Transtibial	35	81
	Transfemoral	5	12
	Through knee	3	7

**Prosthesis design**	Modular	40	93
	Exoskeleton	3	7

**Duration having a prosthesis**	1 – 2 years	20	47
	3 – 5 years	7	16
	6 – 10 years	4	9
	< 10 years	12	30
**Total**		**43**	**100**

* Percentages are rounded.

### Reasons for Prosthesis Non-Use

**Table 2** shows that the most common reason for prosthesis non-use was poor socket fit (22; 51%). The unfortunate death of users constituted 7% (3) of reasons for non-use of lower limb prosthesis.

**Table 2:** Reasons for lower limb prosthesis non-use.

Reasons for prosthesis non-use	Participants (n)	Percentage (%)*
Poorly fitting socket	22	51
Damage of prostheses components	10	23
Exoskeletal prosthesis that could not be repaired	2	5
Prosthesis was shorter than sound limb	1	2
Prosthesis was heavy	1	2
Skin damage and/or residual limb pain when walking	2	5
Residual limb volume changes	2	5
Prosthesis user deceased	3	7
**Total of participants**	**43**	**100**

* Percentages are rounded.

### Component Recycling

**Table 3** shows that forty-two (98%) of participants were in favor of a recycling practice with 91% (39) emphasizing the importance of helping others through recycling of components.

**Table 3:** Participants’ opinions on prosthetic component recycling.

Opinion category	participants (n)	Percentage (%)*
Good or excellent idea to help other people	39	91
Shows that there are parts that can be re-used	1	2
can save costs	1	2
If unused devices are returned willingly	1	2
I have no knowledge about it	1	2
**Total**	**43**	**100**

* Percentages are rounded.

**Table 4** shows that eleven participants (26%) indicated that they could not see any barriers to recycling prosthetic components. Eight participants (19%) cautioned that damaged components could not be re-used.

**Table 4:** Participants’ opinions on barriers of components recycling.

Category	Opinion	Participants (n)	Percentage (%)*
**Barriers**	No barriers	11	26
	Damaged components cannot be re-used.	8	19
	I do not know.	7	16
	Parts may not be suitable for reuse	3	7
	Keeping track of people after fitting the prosthetic device may be difficult.	2	5
	No transport to collect the unused devices	2	5
	People might not know about component reuse may throw away unused devices.	3	7
	People refusing to give back the prosthesis.	2	5
	Contract of agreement to return unused prosthetic devices may be seen as forcing people to comply.	1	2
	Concerns of not getting new components once fitted with recycled ones.	1	2
	Inappropriate technology i.e., energy storing carbon feet contraindicated for household non-active ambulators.	1	2
	Prosthetic parts need to be prepared well before being reused.	1	2
	Refusal to reuse components that were used by a deceased person.	1	2
**Total**		**43**	**100**

* Percentages are rounded.

**Table 5** shows that 22 (51%) of participants indicated that if people understood re-using components can assist others, they will support the practice.

**Table 5:** Factors facilitating component recycling reported by participants.

Category	Opinion	Participants(n)	Percentage (%)*
**Enablers**	If people understand that components reuse will help other people in need of devices.	22	51
	If people can understand that the reuse of parts will save costs.	5	12
	If people can understand that component reuse will help with repairing other peoples’ devices	5	12
	I do not know	3	7
	If people can understand the reuse of components will help reduce waiting time to fit the leg.	4	9
	If people are informed about the components reuse at the hospitals and clinics.	2	5
	Contractual agreement should be based on consultation of users/next of kin to be mutual.	1	2
	Service provider to conduct monthly check-ups on device use	1	2
**Total**		**43**	**100**

* Percentages are rounded.

Forty-one participants (95%) indicated that they would be willing to return the unused prosthesis. One participant who did not want to return the unused prosthesis indicated that the prostheses is used as a spare prosthesis in case the other one breaks. The other participant indicated the desire to be buried with the prosthesis as it feels like part of the body. Among the three next of kins, one returned the prosthesis, another said the prosthesis was buried with the deceased user and the last said the prosthesis was burned during the cultural rite following the death of the user. Three participants (7%) were not willing to sign a contract to return unused prostheses while 39 (91%) were willing and one (2%) was unsure.

Participants indicated that prostheses can be returned in the following ways:

- Return by user or next of kin to prosthetic center: 43 (100%)
- Return to the nearest hospital or clinic: 40 (93%)
- Someone should fetch it at their house: 28 (65%)
- Left at a central point in the community: 5 (12%)

### Number and Type of Prosthetic Components Returned

**Table 6** illustrates that 39 prostheses that were returned with190 components that can possibly be recycled. Commonly, components could be salvaged from modular prostheses (99%), rather than exoskeletal ones. These components included 36-prosthetic feet (19%), 36-foot adaptors (19%), 36-pylons (19%), 36-clamp adaptors (19%), and 36-socket adaptors (19%).

**Table 6:** Distribution of prosthesis type, design, and components.

Type of prosthesis	Prosthesis design	Prostheses (n)	Prosthesis component	Components (n)	Percentage (%)*
**Transtibial prosthesis**	**Modular**	29	Prosthetic foot	29	15
			Prosthetic foot adaptor	29	15
			Pylon/tube	29	15
			Tube clamp adaptor	29	15
			Socket adaptor	29	15
	**Exoskeletal**	2	Prosthetic foot	2	1
**Transfemoral prosthesis**	**Modular**	5	Prosthetic foot	5	3
			Prosthetic foot adaptor	5	3
			Pylon/tube	5	3
			Tube clamp adaptor	5	3
			Prosthetic knee joint	5	3
			Socket adaptor	5	3
**Through knee prosthesis**	**Modular**	2	Prosthetic foot	2	1
			Prosthetic foot adaptor	2	1
			Pylon/tube	2	1
			Tube clamp adaptor	2	1
			Prosthetic knee joint	2	1
			Socket adaptor	2	1
	**Exoskeletal**	1	Prosthesis foot	1	1
**Total number of unused lower limb prostheses**		**39**	**Total number of components for possible recycling**	**190**	**100**

* Percentages are rounded.

## DISCUSSION

This study investigated the factors contributing to the abandonment of lower limb prostheses among persons with lower limb amputation who received a prosthesis in the public healthcare sector of the Eastern Cape Province of South Africa. Findings showed that poorly fitting sockets mainly led to non-use. The study further showed that recovering unused prostheses for components reuse as a strategy for improving accessibility of prosthetic services in resource-constrained settings is feasible. This study provides insight into opportunities for improving prosthetic service delivery while supporting sustainable prosthetic components reuse initiatives.

The findings of this study provide empirical support for adopting lower limb prosthetic component recycling practice in context of the setting. The recovery of 39 unused prostheses and 190 potentially reusable components after dismantling (components must still be tested for structural integrity) demonstrates the feasibility of prosthetic component recycling in the Eastern Cape. Comparable initiatives have reported similarly high rates of second-hand component certification following integrity testing,^38–40^ supporting the generalizability of results to other low-and-middle income country LMIC settings.

The predominance of modular designs among unused prostheses is unsurprising given technological advances and widespread adoption of modular systems due to their flexibility, ease of alignment and repair, interchangeability, and compatibility with component reuse.^41–43^ Modular designs provide more opportunities for recycling than exoskeletal designs as individual components can be dismantled and used individually in different devices.

The high proportion of unused prostheses identified in this study confirms that prosthetic abandonment remains a persistent challenge as described in previous work.^18–22^ Prostheses non-use typically increases several years after initial fitting due to socket discomfort, physiological changes, or death of the user.^18,19,43^ However, in the current study poor socket fit was experienced from early on. This raises concerns about the initial manufacturing and fitting process.

Poor socket fit was the most frequently reported reason for prosthesis non-use. This finding aligns with previous studies highlighting socket fit problems and inadequate follow-up as key contributors to prosthesis abandonment in South Africa,^4,11^ and England.^22^ Residual limb volume reduction especially among first-time users is an expected physiological process that can result in poorly fitting sockets.^44,45^ Fluctuations in limb volume and shape have been consistently associated with prosthesis non-use.^46,47^ Socket replacement rather than providing a new device is typically the recommended clinical response to poor socket fit. However, current study participants received replacement prostheses made entirely of new material and components during the launching of direct socket design project or stopped using the device. The prostheses with the poorly fitting socket were not returned to the place of service provision.

Delays in repairs or replacement might have led to the choice to abandon prostheses and might indicate gaps in follow-up systems. These gaps may be attributed to constrained budgets, high service demand, shortages of skilled personnel,^9,48^ as well as transport and financial barriers faced by users.^49,50^ An addition to the challenges with maintenance, repair and replacement limited follow-up might also mean that devices no longer in use are not identified and recovered. Users and next of kin may also be unaware that unused prostheses can be recycled.

Cultural beliefs surrounding death and burial practices emerged as an important consideration for component recycling that requires further study. In this study the wish to be buried with the prosthesis was mentioned by one user and one next of kin. The extent of this practice is unknown and must be further explored. South Africa has a diverse cultural and ethnic landscape that encompasses varying beliefs regarding the handling of a deceased person’s belongings.^36,51^ In some communities, prostheses may be buried or burned as part of burial rites, reflecting beliefs about respect for the deceased^52^ as also shown in current findings. Therefore, culturally sensitive approaches are essential when implementing prosthetic component recycling initiatives.

Participants’ support for component recycling was consistent with earlier studies demonstrating positive attitudes toward assistive product reuse when safety and functionality are assured.^24^ Participants’ willingness to return unused prostheses appeared linked to their awareness of component shortages and the potential of recycling to improve access and reduce waiting times. This support may reflect users’ experiences of delayed service delivery in public healthcare^53,54^ and the influence of Ubuntu, a philosophy emphasizing shared humanity and collective benefit.^55,56^

Cost considerations further reinforce the perceived value of component recycling. Prosthetic services incur both direct costs (prosthesis manufacture) and indirect costs (travel and maintenance). High travel costs have been identified as a major barrier to prosthetic care in South Africa.^10^ The availability of recycled components may reduce repeated travel and associated expenses, particularly when components require replacement. Given that a subsidized lower limb prosthesis may cost approximately ZAR 7,000 per user in the Eastern Cape,^15^ recycling could mitigate both system-level and user-level financial barriers. Nonetheless, the implementation of recycling practices may introduce additional costs, necessitating careful cost-benefit analyses and inclusion of recovery processes in annual budgets.

Prosthetic components are subjected to mechanical stresses during use,^57^ and wear may occur within months to years.^58^ The use of components intended for household mobility for highly active users in South Africa, may increase the risk of damage and subsequent abandonment.^5^ While damaged components were reported by only 22% of participants it is important that every recovered component be assessed for structural integrity to ensure user safety.^1^ The actual number of components that can be used for recycling and thus the feasibility of recycling can only be determined after structural integrity testing.

Participants’ opinions regarding using contractual agreements for returning unused prostheses further highlight the need for dialogue and mutual understanding. While some viewed contracts as supportive, others perceived them as potentially coercive. The South African Department of Health guidelines on provision of assistive products^34^ includes strategies to recover unused products. In addition to encouraging recycling, these guidelines specify that there should be a standardized contract between the service provider and assistive products user which stipulates that the product remains state property and should be returned if no longer used.^34^ It further states that unused products should be evaluated for recycling, that users should be refunded for returned assistive products that can be re-used and that assistive products should only be recovered if recovery justify the administrative burden.^34^

Any agreement must respect users’ rights to informed decision-making, freedom of expression,^59^ and participation in health policy development.^60^ Balancing legal frameworks, cultural sensitivity, and the principle of Ubuntu is crucial for sustainable implementation of recycling projects. Advocacy, policy development, and public engagement are essential to promote transparent and ethical recycling practices.^61^ Concerns that unused prostheses might be diverted into metal recycling markets further emphasize the need for clear, secure recovery pathways aligned with circular economy principles.^62^ It is important that all relevant stakeholder groups are engaged in ongoing negotiations to develop sustainable prosthetic recycling practices.^27–30^ Stakeholder groups must include political stakeholders to drive policy development and implementation, professionals who provide services such as assessment, measurement and fitting, prosthesis users and representatives from relevant health and rehabilitation forums.^28,29^

### Limitations

The questionnaire used in this study was self-developed and not formally tested for construct validity and reliability, a limitation that can influence the quality of the data negatively. Also, the small sample size reduces the generalizability of findings. Other limitations of this study were that the structured survey limited the participants’ expressions on areas such as the reason for not signing the contract and why some did not choose the option of service provider to fetch unused prosthesis from their homes as an easier way to return it. The data was collected solely from prosthesis users, incorporating perspectives from prosthetic service providers or other stakeholders may be an area for future research. This study focused on lower limb prostheses and cannot inform recycle practice of upper limb prostheses.

### Study Recommendations

Prosthetic service managers and service providers should initiate formal discussions with users or their representatives on implementation of recycling of prosthetic components in the Eastern Cape. These discussions should include 1) the establishment of a tracking system such as scheduling follow-up phone calls or outreach visits, 2) use of a contract of agreement between the user/next-of-kin and service providers, 3) pathway (s) for recovering unused prostheses in consideration of traveling distance and allocation of budget, 4) testing structural integrity of used components, and 5) establishing a designated area to receive unused prostheses, record and store recycled components. There is a need to investigate cultural beliefs around the return and reuse of components that were once used by deceased people.

## CONCLUSION

Participants expressed support for adopting prosthetic component recycling, a view reinforced by the high recovery rate of unused prostheses containing potentially reusable components during the study. Collectively, these findings extend existing knowledge on prostheses abandonment and highlight an opportunity to implement component recycling practices to improve access, reduce waiting times, and lower costs in low-resource settings with high prosthetic service demand. The study findings have important implications for prosthetic service delivery and policy in the Eastern Cape and South Africa, including the development of guidelines for prosthetic component recycling and investment in strengthening follow-up systems for preventive maintenance to reduce abandonment and facilitate recovery of unused prostheses.

## Figures and Tables

**Table:** 

Prosthetic User Survey
Participant Number ……… Thank you for participating in the study. This survey will take 10 to 15 minutes to complete. You are welcome to stop participating at any time. Please complete each question to the best of your knowledge. Please ask me if anything is unclear.
**1)** Please state how old are you in years? **2)** What is your gender? **3)** Please state the date in years and months you were fitted with the prosthesis. **4)** What type of prosthesis you were fitted? **5)** What type of components used in the prosthesis? **6)** What was the cause of amputation? (Please tick the relevant option below) ….. Diabetes Mellitus ….. Peripheral vascular disease ….. Trauma ….. Congenital ….. Cancer ….. Infection ….. Other
**7)** Do you have any other co-morbidities? (Please tick the relevant answers) ….. Respiratory problems ….. Kidney disease ….. Heart failure ….. Stroke ….. Anxiety ….. Panic disorder ….. Vascular disease ….. Other ….. None
**8)** Have you had a stump surgical review after first amputation? (Please tick relevant answer and category of surgical review if yes) Yes ….. Long to medium/short transtibial ….. Transtibial to knee disarticulation ….. Knee disarticulation to transfemoral ….. Transfemoral to hip disarticulation ….. Remain the same amputation level No
**9)** How long have you had your prosthesis? (Please tick the relevant option). ….. 7 – 12 months ….. >12 months
**10)** Can you please tell me the reasons why you cannot use the prosthesis?
**11)** Would you like to be referred for help to assist with the reason (s) provided in question 10? ….. Yes ….. No
**12)** Would you be willing to give back the unused prosthesis to the Prosthetic Centre? ….. Yes ….. No
**13)** If no, can you please explain why not.
**14)** Re-using prosthetic components is a ……… idea. (Please tick your opinion). ….. Good ….. Bad
**15)** Please provide the reason (s) why you think it is a good / bad idea. (Use the choice indicated in question 14 above).
**16)** What will make it easy for you and other people to give an unused prosthesis back to the prosthetic centre? (Please tick all options that you think will be helpful). ….. Taking it back to the prosthetic centre ….. Taking it to the closest hospital ….. Taking it to the community health centre or clinic ….. Leaving it at a central point in the community such as a shop ….. Having someone fetch it from my house ….. Other (please explain)
**17)** Do you think signing a contract that says the prostheses are the property of government and must be returned if not used will help that people return devices that they do not use? ….. Yes ….. No ….. I don’t know
**18)** Please list things that you think will make it difficult to re-use prosthetic components.
**19)** Please list things that you think might help with re-using prosthetic components.
Thank you very much for your time.
